# Mammalian Plakins, Giant Cytolinkers: Versatile Biological Functions and Roles in Cancer

**DOI:** 10.3390/ijms19040974

**Published:** 2018-03-24

**Authors:** Lifang Hu, Zizhan Huang, Zixiang Wu, Arshad Ali, Airong Qian

**Affiliations:** 1 Laboratory for Bone Metabolism, Key Laboratory for Space Biosciences and Biotechnology, School of Life Sciences, Northwestern Polytechnical University, Xi’an 710072, China; hulifang@nwpu.edu.cn (L.H.); louisa@mail.nwpu.edu.cn (Z.H.); wuzx@mail.nwpu.edu.cn (Z.W.); ArshadAli@mail.nwpu.edu.cn (A.A.); 2 Research Center for Special Medicine and Health Systems Engineering, School of Life Sciences, Northwestern Polytechnical University, Xi’an 710072, China; 3 NPU-UAB Joint Laboratory for Bone Metabolism, School of Life Sciences, Northwestern Polytechnical University, Xi’an 710072, China

**Keywords:** plakins, cytoskeleton, cell migration, cell proliferation, cell signaling transduction, cancer

## Abstract

Cancer is a highly lethal disease that is characterized by aberrant cell proliferation, migration, and adhesion, which are closely related to the dynamic changes of cytoskeletons and cytoskeletal-adhesion. These will further result in cell invasion and metastasis. Plakins are a family of giant cytolinkers that connect cytoskeletal elements with each other and to junctional complexes. With various isoforms composed of different domain structures, mammalian plakins are broadly expressed in numerous tissues. They play critical roles in many cellular processes, including cell proliferation, migration, adhesion, and signaling transduction. As these cellular processes are key steps in cancer development, mammalian plakins have in recent years attracted more and more attention for their potential roles in cancer. Current evidence shows the importance of mammalian plakins in various human cancers and demonstrates mammalian plakins as potential biomarkers for cancer. Here, we introduce the basic characteristics of mammalian plakins, review the recent advances in understanding their biological functions, and highlight their roles in human cancers, based on studies performed by us and others. This will provide researchers with a comprehensive understanding of mammalian plakins, new insights into the development of cancer, and novel targets for cancer diagnosis and therapy.

## 1. Introduction

The cytoskeleton, as the important cell scaffold, is a highly organized and interconnected network of filaments consisting of microfilaments (F-actin), microtubules (MTs), and intermediate filaments (IFs). The cytoskeleton is dynamically organized and plays central roles in determining cell shape and numerous cellular functions, including cell polarization, adhesion, migration, proliferation, and intracellular trafficking. All these cellular functions are closely associated with cancer development and progression. Thus, the correct organization of cytoskeleton is critical for cell function. Recently, numerous studies have demonstrated that plakins are versatile giant cytoskeletal crosslinkers and play key roles in regulating cytoskeletal organization and diverse cellular functions [1,2].

Plakins are a family of giant cytolinkers that feature a multidomain structure that enables them to connect the F-actin, MTs, and IFs with each other and to junctional complexes. So far, nine plakins—seven mammalian and two invertebrate—have been discovered [3]. The mammalian plakins are bullous pemphigoid antigen 1 (BPAG1), microtubule actin crosslinking factor 1 (MACF1), plectin, desmoplakin, envoplakin, periplakin, and epiplakin, while the invertebrate plakins are shortstop (shot) in *Drosophila* and VAB-10 in *C. elegans* [3]. Through the integration of F-actin, MTs, and IFs in cytoskeletal networks, plakins are critical in regulating cytoskeletal dynamics and are thus important in regulating cell polarity, adhesion, migration, and proliferation. Moreover, plakins play key roles in mediating signaling transduction, such as wingless and int-1 (Wnt)/β-catenin signaling, extracellular signal-regulated kinases 1/2 (ERK1/2) signaling, and protein kinase B (PKB)-dependent signaling [4,5,6]. Cytoskeletal remodeling and its related cell migration and cell proliferation are key events closely related to cancer development [7]. Additionally, many signaling pathways, such as Wnt/β-catenin and ERK1/2, are critical in cancer development [8,9,10]. Thus, plakins, which are critical modulators for the above events, have attracted more and more attention for their potential roles in cancer.

In this review, we introduce the characteristics of mammalian plakins, summarize the current understanding of their biological functions and highlight their roles in cancer, based on our studies and those of others. This review will provide researchers an increased understanding of mammalian plakins and provide guidance for future research on cancer.

## 2. The Mammalian Plakins

### 2.1. The Isoforms and Tissue Distribution of Mammalian Plakins

Mammalian plakins consist of seven members: BPAG1, MACF1, plectin, desmoplakin, envoplakin, periplakin, and epiplakin [2]. Most of them display isoform diversity and are widely expressed in numerous tissues (Table 1).

**Figure 1:**
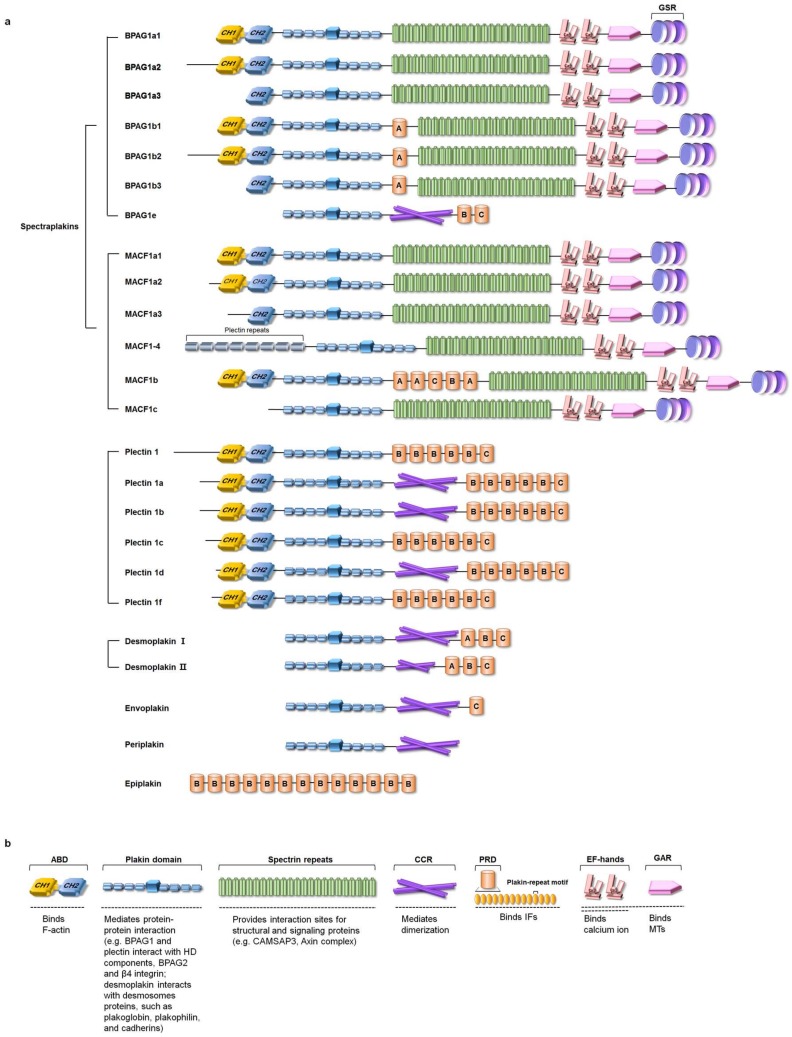
Schematic diagram of the domain structures of mammalian plakins with different isoforms, and the known function of seven main domains. (**a**) The mammalian plakins with a combination of different unique domain structures; (**b**) Seven main types of domain found in mammalian plakins and their known functions. BPAG1: bullous pemphigoid antigen 1; BPAG2: bullous pemphigoid antigen 2; CH: calponin homology; GSR: glycine-serine-arginine; MACF1: microtubule actin crosslinking factor 1; ABD: actin-binding domain; F-actin: actin filaments; HD: hemidesmosome; CAMSAP3: calmodulin regulated spectrin-associated protein 3; CCR: coiled-coil rod; PRD: plakin repeat domain; IFs: intermediate filaments; GAR, GAS2-related protein; MTs, microtubules.

BPAG1, also known as dystonin or bullous pemphigoid antigen 230 (BP230), was originally discovered as a hemidesmosomal protein in patients with the bullous pemphigoid disease [11]. Three major isoforms, named BPAG1a, BPAG1b, and BPAG1e, which are respectively defined as neuronal, muscular, and epithelial isoforms, have been reported [12,13]. Moreover, three additional isoforms from both BPAG1a (BPAG1a1, BPAG1a2, and BPAG1a3) and BPAG1b (BPAG1b1, BPAG1b2, and BPAG1b3), produced by different transcription initiation sites and alternative splicing, have been found. These BPAG1a and BPAG1b isoforms differ in their N-termini and C-termini [12] (Figure 1a). With their different domain structure combinations (Figure 1), BPAG1 isoforms are widely expressed, with some presenting predominant expression patterns in some tissues [14,15] (Table 1).

MACF1 is also named as actin crosslinking factor 7 (ACF7) [16], MACF [17], macrophin [18], and trabeculin-α [19]. It is originally discovered as a member of the actin crosslinker superfamily [16]. Like BPAG1, MACF1 also shows isoform diversity and comprises six isoforms: MACF1a1, MACF1a2, MACF1a3, MACF1-4, MACF1b, and MACF1c [20] (Table 1). These alternative spliced isoforms differ in their domain structures. The MACF1a1 and MACF1a2 isoforms have identical actin-binding domain (ABD) with different 5′ untranslated regions (UTRs), while MACF1a3 only has half an ABD, and MACF1c and MACF1–4 have no ABD in the N-terminal [21,22]. Instead, MACF1–4 contains N-terminal plectin repeats [22]. Compared to MACF1a, MACF1b has extra plakin repeats between the plakin domain and spectrin repeats [23] (Figure 1a). Numerous evidence demonstrates the ubiquitous expression of MACF1, with some isoforms predominantly present in some specific tissues [16,18,22,23,24,25,26] (Table 1).

Plectin was originally discovered as a main constituent of IFs [27]. Composed of an ABD, a plakin domain, a coiled-coil rod (CCR) domain, and plakin repeat domain (PRD) (Figure 1), plectin mediates the interaction of the three main types of cytoskeletal filaments: F-actin, MTs, and IFs [28]. Plectin shows an unusual diversity of isoforms produced by alternative splicing [29]. The plectin isoforms mainly consist of plectin 1, plectin 1a, plectin 1b, plectin 1c, plectin 1d, and plectin 1f, which mainly vary in their N-termini and CCR domain (Figure 1a). With the different N-terminus, plectin isoforms are located at different cellular structures [29] (Table 1). Plectin, since it possesses diverse isoforms and binding sites for all types of IF subunit proteins, is widely expressed in almost all mammalian cell types (e.g., fibroblast, endothelial cells, nerve cells, epithelial cells, smooth muscle cells, monocytes, and keratinocytes) and tissues (e.g., stomach, kidney, small intestine, liver, uterus, urinary bladder, heart, placenta, cornea, skin, foreskin, and eyelid skin), while some isoforms are predominant in the skeletal muscle [30,31,32] (Table 1).

Desmoplakin was identified as a critical component of desmosomes in epidermal cells and has two alternatively spliced isoforms, desmoplakin I and desmoplakin II [33,34]. Desmoplakin I and desmoplakin II both comprise an N-terminal region consisting of a plakin domain and a CCR domain, as well as a C-terminal region containing three plakin repeat domains (PRDs) (Figure 1a). However, desmoplakin II contains a shorter CCR domain compared with desmoplakin I [35] (Figure 1a). As one important component of desmosomes that provide strong adhesion between cells, desmoplakin I and II are present in all epithelia, while desmoplakin II is not detected in cardiac muscle [36] (Table 1).

Envoplakin and periplakin were first found to be components of the cornified envelope in the terminal differentiation of epidermal keratinocytes [37]. Further studies demonstrate that envoplakin and periplakin colocalize with desmoplakin at desmosomes, as well as on keratin filaments in the human epidermis [38,39]. Envoplakin is homologous to desmoplakin, BPAG1 and plectin, while periplakin is also one component of desmosomes. Envoplakin and periplakin both consist of the N-terminal plakin domain, central CCR and C-terminal L subdomain, while envoplakin has an additional C type PRD in its C-terminus (Figure 1a). Through the CCR, the envoplakin and periplakin can form heterodimers [39,40]. Without isoforms being reported, envoplakin and periplakin mainly exist in keratinizing and nonkeratinizing stratified squamous epithelia, but not in most simple epithelia or nonepithelial cells [38,39] (Table 1).

Epiplakin was originally discovered as a 450 kD human epidermal autoantigen that reacted with the serum from a patient with a subepidermal blistering disease [41]. Further molecular cloning of the epiplakin protein reveals that it has a molecular mass of 552 kD [42]. Epiplakin is an unusual plakin due to its unique domain structure that contains only 13 B-type PRDs but lacks an N-terminal plakin domain and CCR domain [42] (Figure 1a). Like other plakins, epiplakin is broadly expressed in many tissues [42,43,44] (Table 1).

### 2.2. Domain Structure of Plakins

The mammalian plakins, some of which have isoforms, are composed of different combinations of varied domains that mainly include the ABD, plakin domain, spectrin repeats, CCR, PRD, EF-hand, and growth arrest specific 2 (GAS2)-related protein (GAR) domain (Figure 1). Specifically, BPAG1 and MACF1 are also grouped as “spectraplakins” because they have both spectrin repeats and a plakin domain [45]. Each domain has its specific function for conferring plakins with the ability to interact with F-actin, MTs, and IFs, as well as cellular junctional proteins and signaling molecules (Figure 1b).

#### 2.2.1. Actin-Binding Domain (ABD)

ABDs are found in BPAG1, MACF1, and plectin, and they are located at the N-terminal (Figure 1a). Two calponin homology (CH) domains, CH1 and CH2, are arranged in tandem to form the ABD (Figure 1b). Although the CH1 domain can bind actin by itself and CH2 also has a weaker binding affinity, the tandem organization pattern of CH1 and CH2 greatly increases the binding affinity for F-actin [46,47,48]. Further crystal structure analysis of the F-actin-binding domain of MACF1 reveals that the ABD adopts a closed conformation with extensive intramolecular contacts between the CH1 and CH2 domains [49]. Moreover, there is a key tyrosine-phosphorylation site within the ABD of MACF1, and the phosphorylation of ABD by Src/focal adhesion kinase (FAK) is essential for the F-actin-binding of MACF1 [49].

Thus, the ABD confers plakins with a capacity to bind F-actin and to regulate cytoskeletal dynamics. For example, the ABD of MACF1 mediates the interaction between MACF1 and F-actin, and coordinates the MTs/F-actin dynamics to regulate directed cell migration [50]. Although the tandem organization of two CH domains exerts high binding affinity for F-actin, some isoforms of BPAG1 and MACF1 produced by alternative splicing have only the CH2 domain or no CH domain [13,20,51,52] (Figure 1a). The specific function of this alternative splicing is still unknown and requires further study.

#### 2.2.2. Plakin Domain

The plakin domain is one specific feature of the plakin family, and all plakins except epiplakin possess a plakin domain characterized by a high α-helical content. Both protein structure analysis and crystallography determination have demonstrated that the plakin domain consists of two pairs of spectrin repeats and an inserted Src-Homology 3 (SH3) domain [53,54] (Figure 1b).

The plakin domain provides an important platform for protein–protein interactions and makes plakins interact with components of specialized cell junctions, such as hemidesmosomes (HDs, which are specific cell-matrix junctions) and desmosomes (cell–cell junctions). The plakin domain facilitates the interaction of BPAG1 and plectin with HD components, BPAG2 and β4 integrin, in order to strengthen the stable adhesion of epithelial cells to the extracellular matrix [55,56,57]. Moreover, the plakin domain of desmoplakin has been shown to interact with desmosomes proteins, such as plakoglobin, plakophilin, and cadherins [58].

#### 2.2.3. Spectrin Repeats

Spectrin repeats present one main domain of the spectraplakins BPAG1 and MACF1 (Figure 1a). Each spectrin repeat is a 106–122 amino acid segment that folds into three α-helices. These α-helices form an antiparallel triple-helical coiled coil [59,60]. These spectrin repeats compose an extended rod-like structure, which is located between the N- and C-terminal functional domains and acts as a spacer region to confer proteins with flexibility.

Spectrin repeats provide important interaction sites for various structural and signaling proteins [61]. The spectrin repeats of MACF1 bind to the MT minus-end-binding protein CAMSAP3 (calmodulin regulated spectrin-associated protein 3), and mediate the interaction between MACF1 and CAMSAP3 [62]. Moreover, the spectrin repeats mediate the participation of MACF1 in Wnt/β-catenin signaling by associating with a protein complex containing Axin, adenomatous polyposis coli (APC), β-catenin, and glycogen synthase kinase 3β (GSK-3β) [4].

#### 2.2.4. Coiled-Coil Rod (CCR)

The CCR domain exists in most mammalian plakins, including BPAG1e, plectin, desmoplakin, envoplakin, and periplakin [1]. The CCR domain is composed of heptad repeats and generally presented between the plakin domain and the PRD (Figure 1a).

Evidence shows that the CCR domain mediates the dimerization of plakins [63]. However, the function of the CCR domain has not been extensively studied and requires further investigation.

#### 2.2.5. Plakin Repeat Domain (PRD)

PRD is a distinctive hallmark of the plakin family. It is a globular domain composed of 4.5 copies of plakin-repeat motif with each repeat motif containing 38 amino acids [64] (Figure 1b). PRDs are classified as three types, A, B, and C [35,64], and are found at the C-terminus of most plakins and at the internal region of some spectraplakin isoforms. However, epiplakin is solely composed of the B-type PRD (Figure 1a).

PRDs are responsible for binding to various IFs, as demonstrated in BPAG1 [65], plectin [66], desmoplakin [64], envoplakin [67], and epiplakin [68]. Choi et al. solved the crystal structures of B- and C-type PRDs of desmoplakin and found a conserved basic groove on the domain, which could serve as an IF-binding site [64]. Fogl et al. further reported the mechanism by which envoplakin bound to IFs by solving the envoplakin PRD crystal structure [67]. They found that a basic groove on the PRD could accommodate acidic patches within IF proteins. In addition, the residues within the groove mediated the targeting of envoplakin to vimentin and keratin IFs [67]. These studies demonstrate that PRDs confer plakins with the ability to link IFs. However, the PRDs in MACF1b have been shown to target MACF1b to the Golgi complex [23]. This suggests that the specific role of different PRD types (A, B, and C) and their different combinations may require further study.

#### 2.2.6. EF-Hand and GAS2-Related Protein (GAR) Domain

Like spectrin repeats, the EF-hand and GAR domain is present in spectraplakins BPAG1 and MACF1 (Figure 1a). There are two EF-hand motifs followed by a GAR domain at the C-terminus of the spectraplakins [69]. The GAR domain is named after the growth arrest specific 2 (Gas2) and contains ~57 amino acids. The crystal structure analysis of the hACF7 EF1-EF2-GAR module reveals that the EF1-EF2 and GAR domains are connected by a flexible linker [70].

The EF1-EF2 domain functions in calcium-binding, and each EF-hand motif presents as an EF β-scaffold with two bound Ca^2+^ ions that straddle an N-terminal α helix [70]. The GAR domain is responsible for binding to and stabilizing MTs [21,70]. While the EF1-EF2 domain is not sufficient for MT binding, the GAR domain enhances EF1-EF2-MT engagement [70]. Although the EF-hand motifs of MACF1 have no effect on MACF1 interacting with MTs [21], the EF-hand motifs of BPAG1 have been shown to play an important role in BPAG1-binding MTs [71]. By adopting the eukaryotic expression vector pFLAG-MACF-GAR-GSR that encoded a FLAG-tagged MACF1 C-terminal protein without the EF-hand motifs to transfect the COS-7 cells, Sun et al. found that the overexpressed MACF1 GAR-GSR proteins colocalized perfectly with the MT network, which was similar to the result obtained with the entire MACF1 C-terminal protein (containing EF-hand motifs). This finding indicates that the EF-hand motifs of MACF1 have no effect on the interaction between MACF1 and MTs [21]. However, Kapur et al. found that mutations of the EF-hands abolished the switch between the MT plus end interaction and the MT lattice binding of BPAG1n4, suggesting the importance of EF-hand motifs in BPAG1-binding MTs [71].

## 3. Biological Functions of Mammalian Plakins

### 3.1. Mammalian Plakins in Cell Migration

Cell migration is a multistep process consisting mainly of four coordinated steps: (1) protrusion of a cell’s leading edge; (2) adhesion to the substrate; (3) contraction of the cell body; and (4) the turnover of focal adhesions (FAs) and retraction. Each step contains the dynamic changes of the F-actin and MTs cytoskeleton. Both F-actin and MTs are polymer structures that can remodel themselves to facilitate cell migration. In cells, actin exists either in monomeric (G-actin) or polymeric forms (F-actin). F-actin is comprised of double helical polymers that are formed by G-actin. MTs consist of α/β-tubulin heterodimers that selfassociate into polymers. F-actin and MTs show structural polarity with two ends, a plus end and a minus end. They undergo fast assembly and disassembly dynamics during cell migration [72,73]. Evidence shows that F-actin plays a key role in stabilizing and guiding MTs on their trek from the microtubule organizing center (MTOC) to the cell periphery [74,75]. Moreover, Salmon et al. have reported that MTs grow along F-actin bundles that guide the movement and organization of MTs during cell migration [76]. They suggest that the guidance of MTs along F-actin bundles may be mediated by some MTs/F-actin crosslinking factor [76].

**Figure 2:**
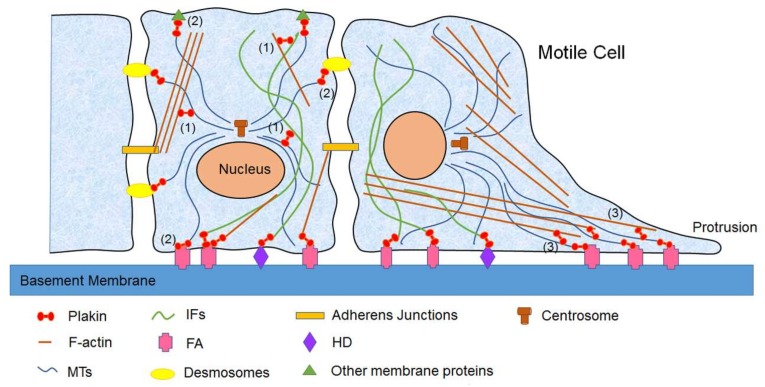
Schematic illustration of the regulatory role of mammalian plakins in cell migration and cytoskeletal dynamics. Plakins regulate cytoskeletal dynamics and cell migration by crosslinking different cytoskeletal networks and adhesion complexes. As giant cytolinkers, plakins show key roles in at least three different cellular processes, as indicated in this figure. (**1**) Plakins crosslink all three cytoskeletal elements: F-actin, MTs, and IFs; (**2**) Plakins connect the cytoskeleton with adherens junctions or membrane proteins; (**3**) Plakins guide MTs along F-actin to target FAs to regulate cell migration.

Plakins, as important crosslinkers for cytoskeleton, show a critical role in regulating cell migration by orchestrating the organization and dynamics of F-actin, MTs, and IFs, as well as the interaction between the cytoskeleton and junctional complexes (Figure 2).

The knockout of *BPAG1* inhibits the epidermal cell migration by severing the attachment between keratin IFs and HDs [77]. Moreover, the knockdown of BPAG1a and BPAG1b causes a specific decrease in the directness of cell migration in myoblasts possibly by increasing dispersal of the Golgi apparatus, which influences MT orientation toward the cell’s leading edge [78]. BPAG1e deficiency leads to a loss of cell polarity and an aberrant cell migration in keratinocytes, through the reduction of the β4 integrin-mediated activity of Rac1 and cofilin [79]. In addition, a BPAG1e deficiency causes the rearrangement of the actin cytoskeleton and the changes in the focal contact protein distribution, which work together with Rac1 and cofilin to form lamellipodia [79]. However, the keratinocytes isolated from patients carrying homozygous nonsense mutations in the *BPAG1e* gene display increased cell migration, along with abnormal levels of keratin-14, and β4 and β1 integrins [80]. Further BPAG1e knockdown studies of normal keratinocytes in vitro failed to recapitulate the above changes in vivo, suggesting that the incomplete depletion of BPAG1e is not sufficient to induce the in vivo phenotype [80]. These studies suggest that BPAG1 is needed to regulate cell migration. Moreover, it is demonstrated that different BPAG1 isoforms exert either promotion or inhibition effects on cell migration possibly via the following mechanisms: (1) mediating the connections between keratin IFs and HDs; (2) maintaining the Golgi apparatus structure that influences MT orientation toward the leading edge of the cell; (3) mediating β4-integrin-mediated activation of Rac1 and cofilin; and (4) controlling adhesion stability.

MACF1 is critical in regulating cell migration because it coordinates the dynamics of F-actin, MTs, and FAs and mediates GSK-3β signaling [81,82,83]. By binding to both F-actin and MTs, MACF1 guides MTs along F-actin to target FAs, thus regulating epidermal cell migration [81]. A loss of MACF1 suppresses cell migration by impairing the targeting of MTs to FAs and by stabilizing FA-actin networks in epidermal cells [81]. An MACF1 absence also inhibits cell migration by impairing the association of MACF1 with GSK-3β signaling in both skin stem cells and neurons [82,83]. An abnormal interneuron migration was also observed in MACF1 conditional knockout mice [84]. In addition, MACF1 regulates cell migration by being recruited by some molecules, such as ErbB2 and engulfment and motility (ELMO) [85,86].

Plectin shows the capacity to bind to F-actin, IFs, and MTs, acting as a regulator of the cytoskeleton dynamics [87,88,89]. Plectin deficiency impairs stress fiber and FA dynamics by increasing the number of the actin stress fibers and FAs [87], inhibits vimentin network dynamics and the stepwise formation of stable IFs [88], and decreases MT dynamics [89]. Additionally, plectin mediates the crosstalk between vimentin and actin networks, and plectin deficiency destroys the shape of adherens junctions and tight junctions [90]. By regulating these cytoskeletal dynamics and cell adhesions, plectin regulates cell migration in a cell-type-dependent manner. A loss of plectin decreases cell migration in lymphocytes [91] but increases cell migration in keratinocytes [92]. Moreover, plectin deficiency increases cell migration by activating Rac1 and FAK in normal human hepatic cells [93]. This indicates plectin’s cell-type-dependent effects on cell migration, and this effect requires further verification in more cell types.

Furthermore, periplakin and epiplakin have also been demonstrated to regulate epithelial cell migration by controlling cytoskeleton reorganization or the expression of adhesion and cytoskeletal proteins [94,95]. Moreover, periplakin has been shown to regulate epithelial migration by interacting with plectin [96], suggesting that crosstalk exists between different mammalian plakins and that they may work together to regulate cell migration.

### 3.2. Mammalian Plakins in Cell Proliferation

Evidence has shown the involvement of mammalian plakins in cell proliferation, especially in cancer cells (this is discussed in further detail in Section 4), although extensive studies are needed.

Our studies show that MACF1 regulates cell proliferation of osteoblasts. By adopting a stable MACF1-knockdown MC3T3-E1 osteoblastic cell line, we found that the knockdown of MACF1 significantly inhibited cell proliferation by disrupting the normal organization of F-actin and MTs and by inducing the cell cycle arrest at the S phase [97]. In normal cells, F-actin was distributed at the cell periphery or passed through the cytoplasm as rope-like structures, and MTs radiated from the MTOC to the cell membrane and presented as tortuous filaments. Meanwhile, in the MACF1-knockdown cells, the F-actin filaments were mainly localized at the cell periphery, and MTs became straighter filaments with a loss of the MTOC. The disorganization of F-actin and MTs may cause defective cytokinesis that results in the cell cycle arrest at the S phase, which leads to decreased cell proliferation in MACF1-knockdown cells [97]. We further determined the effect of MACF1 on cell proliferation by transfecting the MC3T3-E1 osteoblastic cells and MACF1-knockdown cells with MACF1 overexpression plasmid, respectively. The results showed that the overexpression of MACF1 increased cell proliferation in MC3T3-E1 cells and partially rescued the decrease of cell proliferation in MACF1-knockdown cells [98]. Thus, our findings indicate the promotion effect of MACF1 on cell proliferation. However, Fuchs’ group reported that a lack of MACF1 did not cause a significant decrease in cell proliferation nor defective mitosis in epidermal and endodermal cells [50,81,82]. These different findings may be due to the different cell types, suggesting that more studies of different cell types are needed.

Plectin has been indirectly shown to regulate cell proliferation by forming complexes with integrin β4 [99]. Jeon et al. found that the reduction of the integrin β4/plectin complex induced by glucosamine promoted cell proliferation in mouse embryonic stem cells. They further revealed that the decrease of the β4/plectin complex was due to the increase of protein kinase C (PKC) phosphorylation, as well as specificity protein 1 (Sp1) and Snail1 glycosylation [99]. Although little is known about the role of plectin in cell proliferation, this finding paves a way for further studies.

More recently, Kokado et al. reported that the knockdown of epiplakin in corneal epithelial cells significantly accelerated cell proliferation, suggesting a negative regulatory role of epiplakin on cell proliferation [95].

The above findings on MACF1, plectin, and epiplakin illustrate the function of mammalian plakins in cell proliferation.

### 3.3. Mammalian Plakins in Cell Signaling

Besides crosslinking F-actin, MTs, IFs, and junctional complexes, plakins also interact with signaling proteins and possess phosphorylation sites, thus participating in cell signaling transduction to regulate cellular functions, such as cell migration, cell proliferation, and cell differentiation.

BPAG1e has been demonstrated to be a scaffold for Rac1/cofilin signal transduction [79]. The knockdown of BPAG1e dramatically decreased the amount of Rac1 associating with β4 integrin, as well as the activity of both Rac1 and cofilin [79]. By determining the activation of the Rac1/cofilin signal, BPAG1e is essential for the efficient regulation of keratinocyte polarity and migration.

MACF1 possesses phosphorylation sites for GSK-3β at the C-terminus and mediates GSK-3β signaling [82,83]. Moreover, MACF1 is essential for Wnt/β-catenin signaling transduction by interacting with the Axin complex that contained Axin, GSK-3β, β-catenin, and APC, and by translocating the complex from cytoplasm to the cell membrane [4]. Upon Wnt stimulation, MACF1 forms a complex in the cytosol with Axin, GSK-3β, β-catenin, and APC, and translocates the Axin complex (except for APC) to the cell membrane, where they interact with low-density lipoprotein receptor 5/6 (LRP5/6). Subsequently, GSK-3β is inactivated, and β-catenin is released and translocated into the nucleus to activate target genes. However, the loss of MACF1 impairs the Axin complex translocation, inhibits the nuclear translocation of β-catenin, and thus suppresses the downstream T-cell factor (TCF)/β-catenin-dependent transcriptional activation [4]. Our studies further reveal that MACF1 promotes osteoblast differentiation and bone formation by activating β-catenin/TCF1-dependent osteoblastic genes expression [100,101]. These findings indicate the importance of MACF1 in mediating cell signaling transduction.

Like MACF1, plectin also has phosphorylation sites in its C-terminal region and serves as a target for protein kinases, such as p34^cdc2^ kinase and protein kinase A (PKA) [102,103]. The phosphorylation of plectin by PKA or mitogen-activated protein kinase-interacting kinase 2 (MNK2) attenuates the interaction between plectin and IFs, thus remodeling IF networks and the cell function [104]. More recently, Matsubara et al. found that plectin was phosphorylated by tyrosine kinase Src to regulate the actin ring formation and osteoclast differentiation [105]. Moreover, plectin modulates several signaling pathways, such as ERK1/2 signaling and adenosine 5′-monophosphate-activated protein kinase (AMPK) signaling [5,106]. These indicate the key role of plectin in cell signaling, where it either acts as a substrate for kinases or modulates the signaling by itself.

Desmoplakin and periplakin also function in cell signaling transduction. Similar to MACF1 and plectin, desmoplakin participates in cell signaling through either being post-translationally modified by phosphorylation or by directly regulating the signaling pathway. Desmoplakin is phosphorylated by glycogen synthase kinase 3 (GSK3) to control desmoplakin-cytoskeleton dynamics, and it also activates AKT-Wnt/β-catenin signaling [107,108]. Periplakin has been shown to act as a scaffold for the modulation of protein kinase B (PKB)-dependent signaling [6]. In addition, it has been identified as the first protein to modify the melanin-concentrating hormone-1 (MCH-1) receptor-mediated signaling transduction [109]. By binding to the MCH-1 receptor, periplakin reduces the capacity of the receptor to activate G proteins and hence initiate signal transduction [109].

All of the above findings show the importance of mammalian plakins in cell signaling transduction. They are similar in that they either act as substrates for post-translational modification or regulate signaling pathways by themselves.

## 4. Mammalian Plakins in Human Cancer

### 4.1. The Role of Mammalian Plakins in Cancer

With versatility in biological functions closely related to cancer, the role of mammalian plakins in various cancers has recently been shown, as discussed below.

#### 4.1.1. Bullous Pemphigoid Antigen 1 (BPAG1) in Cancer

BPAG1 has recently been linked to cancer. The decrease of BPAG1 was observed in invasive breast cancer cells, nasopharyngeal carcinoma cells, and metastatic prostate cancer [110,111,112]. Since BPAG1 is a major component of HD that mediates the attachment of epithelial cells to the basement membrane, the decrease of BPAG1 may facilitate cancer cell invasion and metastasis. However, Chaudhari et al. found that BPAG1e positively regulated the cell motility of oral squamous cell carcinoma (OSCC)-derived cells [113]. The knockdown of both BPAG1e and plectin, which are two HD linker proteins, inhibits the OSCC-derived cell motility, invasion, and tumorigenicity, possibly by upregulating the N-Myc downstream regulated gene 1 (NDRG1) [114]. These inconsistent findings indicate that BPAG1 may function differently in different cancers or that there are cancer-specific BPAG1 isoforms. The discovery that the alternative splicing of the *dystonin* (*DST*) gene (encoding for BPAG1) occurs in head and neck squamous cell carcinoma (HNSCC) provides a basis for the existence of cancer-specific BPAG1 isoforms [115]. Based on current findings (Table 2), more studies are needed to unravel the role of BPAG1 in cancer, as well as related mechanisms.

#### 4.1.2. Microtubule Actin Crosslinking Factor 1 (MACF1) in Cancer

MACF1 has recently received increasing attention for its role in various cancers (Table 2). It was identified as a cancer biomarker candidate for lung cancer by an integrated genome-scale co-expression network [116]. The upregulation of MACF1 was detected in lung adenocarcinoma with a connection to metastasis and migration [116]. Further experiments directly validated the high level of MACF1 in several lung cancers, and the knockdown of MACF1 inhibited the reproductivity of solid tumors [117]. MACF1 was also linked to breast cancer by a genome-wide DNA methylation profiles analysis and was speculated to regulate cell motility [118]. Del Valle et al. further reported the different levels of MACF1 in stromal cells in different tumor sites of breast cancer patients [119]. Moreover, the mutation of *MACF1* in renal cell carcinoma [120], endometrial cancer [121], and colon cancer [122] has been detected by whole-exome sequencing and targeted gene sequencing. It has also been shown that the *MACF1* mutation was correlated with Wnt/β-catenin signaling in cancer [120,121,122]. While most findings indirectly show the role of MACF1 in cancers, Quick’s group was the first to provide direct evidence of MACF1’s function in glioblastoma [123]. They found that MACF1 was predominately expressed in glioblastomas but not in either normal brain tissue or lower-grade brain tumors. Further suppression of MACF1 in glioblastoma cells inhibited cell proliferation and migration associated with reduced Axin1 and β-catenin, which are Wnt signaling mediators [123]. As MACF1 is critical in mediating the Wnt signaling pathway [4], it is suggested that MACF1 may function in cancers through Wnt signaling. In addition, MACF1 has also been shown to be a target of microRNA in both hepatocellular carcinoma and malignant tumors [124,125]. All these findings demonstrate that MACF1 is a promising target for cancer diagnosis and therapy [126].

#### 4.1.3. Plectin in Cancer

Plectin has been identified as an excellent biomarker for pancreatic intraductal papillary mucinous neoplasms (IPMNs) and as a novel biomarker for pancreatic cancer [127,128]. High levels of plectin have also been observed in HNSCC [129], colon carcinoma cells [130], and high-metastatic bladder cancer cells [131], showing promotion effects on cancer cell migration, invasion, and metastasis. The suppression of plectin inhibits cell migration and invasion in both HNSCC and colon carcinoma cells [129,130]. In HNSCC, plectin has been shown to promote the migration and invasion of HNSCC cells through activation of ERK1/2 [129], while in colon carcinoma cells plectin is targeted to podosome-like adhesions to regulate cell migration and invasion in an isoform-specific manner [130]. Moreover, in high-metastatic bladder cancer, plectin plays a key role in anchoring invadopodia to IF and in stabilizing invadopodia, which is critical for cancer cell invasion [131]. This all demonstrates plectin’s promotion effect on cancer cell migration and cancer progression. Chaudhari et al. also found that the inhibition of plectin reduced cell migration, invasion, and tumorigenicity in OSCC-derived cells [114]. However, a recent study showed a low expression of plectin in liver cancer cells, accompanied by a high cell migration rate, suggesting an inhibitory effect of plectin on liver cancer cell migration [132]. As plectin regulates cell migration in a cell-type-dependent manner, as discussed above (Section 3.1), it may also regulate cancer cell migration in this way. In any case, plectin is a highly reliable biomarker and a potential target for various cancer diagnoses and therapies (Table 2).

#### 4.1.4. Desmoplakin in Cancer

Desmoplakin has been shown to be a tumor suppressor with decreased expression observed in several cancers. Decreased desmoplakin has been detected in oral cancer, including OSCC and metastatic oropharyngeal cancer, and has been shown to be a useful marker for evaluating changes in tissue morphology, as well as the risk of distant metastasis formation [133,134]. Interestingly, the desmoplakin II isoform may play a specific function in oral carcinogenesis because its expression is only detected in tumors associated with a poor clinical outcome [134]. Furthermore, Yang et al. provide direct evidence that desmoplakin functions as a tumor suppressor [135]. They detected a downregulation of desmoplakin in lung cancer and found that an overexpression of desmoplakin inhibited lung cancer cell proliferation, migration, and invasion and that it increased the sensitivity of lung cancer cells to anticancer-drug-induced apoptosis through the inhibition of Wnt/β-catenin signaling [135]. All of this shows desmoplakin’s role as a tumor suppressor, with potential implications for clinical applications.

#### 4.1.5. Other Mammalian Plakins in Cancer

More recently, envoplakin and periplakin, functioning as epidermal barrier components, have also attracted attention in cancer development (Table 2). Cipolat et al. found that mice that were triply deficient in envoplakin, periplakin, and involucrin (EPI−/− mice) were highly resistant to skin tumor development [136], indirectly suggesting a positive role of envoplakin and periplakin in skin cancer development. Tonoike et al. also showed that periplakin knockdown reduced cell movement and attachment via a suppression of the phosphatidylinositol 3′ kinase (PI3K)/Akt axis in pharyngeal squamous cancer cells [137]. Interestingly, a downregulation of periplakin was observed in urinary bladder cancer [138], esophageal squamous cancer [139,140], and colon carcinoma [141], pointing to periplakin’s role as a cancer suppressor. The cancer-suppressive role of periplakin was further confirmed by Li et al. [141]. They reported that an overexpression of periplakin reduced cancer cell migration and invasion, induced a G1/G0 cell cycle arrest, and inhibited colon cancer cell proliferation by suppressing the activation of ERK1/2 and the expression of proliferating cell nuclear antigen (PCNA). Meanwhile, a periplakin knockdown showed contrary effects [141]. Moreover, Otsubo et al. indicated that the decreased periplakin was due to DNA hypermethylation, which contributes to the metastatic phenotype of cancer [140]. Additionally, the dislocation of periplakin from the cell–cell boundaries to the cytoplasm may promote cell invasion and cause tumor development [139]. All of this points to periplakin being a cancer suppressor for numerous cancer types, although there are some contrary findings. Compared to other plakins, epiplakin’s function and role in cancer are still largely unknown. As a plakin that is solely composed of PRD, and that is therefore unusual, epiplakin is only found to be downregulated in pancreatic cancer [43] (Table 2). Its function and mechanisms in cancer require further investigation.

**Figure 3:**
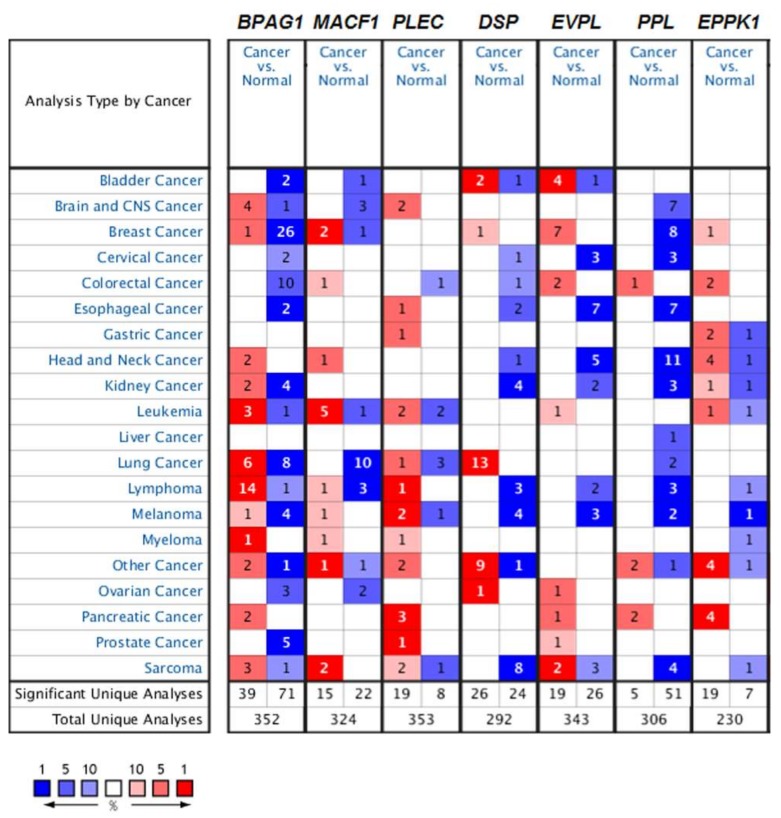
The expression levels of mammalian plakins across different cancers analyzed by Oncomine. The differences in expression levels of the genes between cancer and normal tissue are summarized in the graph. The graph demonstrates the numbers of datasets with statistically significant mRNA overexpression (red cell) or downexpression (blue cell) of the target gene. The number of unique analyses satisfies the threshold settings: *p* value is 0.01, fold change is 2, gene rank is top 10%, data type: mRNA. The gene rank is indicated by the color depth in the cells. *PLEC*: plecitn; *DSP*: desmoplakin; *EVPL*: envoplakin; *PPL*: periplakin; *EPPK1*: epiplakin.

These reports demonstrate the critical role of mammalian plakins in various human cancers (Table 2). Moreover, we investigate the expression levels of mammalian plakins in a broader set of cancers by using the Oncomine, a web-based microarray database (http://www.oncomine.org). As shown in Figure 3, there are 352, 324, 353, 292, 343, 306 and 230 unique analyses for BPAG1 (*BPAG1*), MACF1 (*MACF1*), plectin (*PLEC*), desmoplakin (*DSP*), envoplakin (*EVPL*), periplakin (*PPL*), and epiplakin (*EPPK1*), respectively. For BPAG1 expression, a total of 110 studies show the significant difference between cancer and normal tissues, across all cancer types. Most of the datasets show decreased mRNA levels of BPAG1 in cancer tissues, compared to normal tissues. The most notable cancer types are breast, prostate, melanoma, and colorectal cancer, in which BPAG1 mRNA levels are significantly reduced in cancer cases in 26, 5, 4, and 10 unique analyses, respectively (Figure 3). The result is consistent with findings on breast and prostate cancer reported by Schuetz et al. and Vanaja et al. [110,112] (Table 2). Similar to BPAG1, in most of the datasets, MACF1 mRNA levels are significantly decreased in cancer as opposed to normal tissues (Figure 3). Interestingly, contrary to the findings of Bidkhori et al. [116,117] (Table 2), 10 unique analyses show that the MACF1 mRNA levels are significantly reduced in lung cancer (Figure 3). This difference may require further study. Downexpressions of *EVPL* and *PPL* can also be found in the majority of datasets (26 for *EVPL* and 51 for *PPL*) across the cancer types. For *PLEC* and *EPPK1*, 19 datasets show increased expression in cancers, while only 8 datasets and 7 datasets demonstrate decreased expression of *PLEC* and *EPPK1* in cancers, respectively (Figure 3). *DSP* displays significant overexpression in cancers in 26 datasets and downexpression in 24 datasets. Overexpression and downexpression of mammalian plakins occur across the multiple cancer types, indicated by Oncomine data. However, the expression trend of mammalian plakins in some cancer type is contrary to the literature reports (Table 2, Figure 3). These differences require further determination.

### 4.2. Mammalian Plakins as Potential Targets for Cancer Therapy

The above findings demonstrate the importance of mammalian plakins in cancer, suggesting them as potential targets for cancer therapy. Several evidences show the potential of mammalian plakins as promising targets for cancer therapy, though there is no drug or pharmacological agent that specifically targets them at present. Lorch et al. have reported that treatment with the proteasome inhibitor bortezomib in OSCC results in significant increased desmoplakin and reduced cell migration [142]. As the downregulation of desmoplakin has been found in OSCC [133] (Table 2), the finding of Lorch et al. suggests desmoplakin as a potential target for cancer therapy. More recently, Quick, by using the Research Collaboratory for Structural Bioinformatics (RCSB) protein data bank, has identified several anticancer drugs that interact with BPAG1, MACF1, and plectin [143]. Ibrutinib is one common ligand for both BPAG1 and MACF1, and is used clinically for the treatment of lymphoma and leukemia [144,145]. PP121, showing antitumorigenic effects on esophageal cancer and anaplastic thyroid carcinoma by perturbing PI3K and mammalian target of rapamycin (mTOR) kinase activities [146,147], is identified as an MACF1 ligand. Moreover, plectin is determined to be a substrate for sparsomycin, pactamycin, amicoumacin A, and omacetaxine mepesuccinate, all of which show antitumorigenic effects in leukemia, HNSCC, breast cancer, or lung cancer [148,149,150,151].

Therefore, mammalian plakins play key roles in various human cancers and could be novel biomarkers and promising targets for cancer diagnosis and therapy.

## 5. Conclusions and Perspectives

Cancer is one of the most lethal diseases in the world. It is characterized by an uncontrolled cell proliferation, migration, and adhesion, which are closely related to the dynamic changes of the cytoskeleton and cytoskeletal-adhesion. Furthermore, the uncontrolled cell proliferation, migration, and adhesion will cause cell invasion and metastasis, which make cancer malignant and lethal. Unraveling the underlying mechanisms of aberrant cell proliferation, migration, and adhesion is therefore important not only for understanding cancer development but also for providing new targets for cancer diagnosis and therapy.

Plakins are a family of giant cytolinkers that play important roles in cell proliferation, migration, and signaling transduction by modulating the dynamics of F-actin, MTs, IFs, and FAs and by regulating signaling pathways. There are seven mammalian plakins: BPAG1, MACF1, plectin, desmoplakin, envoplakin, periplakin, and epiplakin. They are broadly expressed in numerous tissues. Most of them (BPAG1, MACF1, plectin, and desmoplakin) have isoforms, and all of them have multidomain structures that enable them to connect with different cytoskeletal and signaling molecules. Because they possess various isoforms, structures, and tissue distributions, mammalian plakins are critical in maintaining normal cell proliferation, migration, and signaling transduction. In regulating these cellular processes, mammalian plakins either coordinate the cytoskeletal-adhesion dynamics (e.g., cytoskeleton-HDs) or adjust signaling pathways, such as GSK-3β signaling, Wnt/β-catenin signaling, Rac1/cofilin signaling, ERK1/2 signaling, and AMPK signaling.

Mammalian plakins show their significance in human cancers by showing versatility in their regulation of cell proliferation, migration, and signaling transduction, key steps for cancer development, and by showing potential to be targets for anticancer drugs. It has been demonstrated that the altered expression or mutation of mammalian plakins is closely related to numerous cancers, such as breast cancer, prostate cancer, oral cancer, lung cancer, endometrial cancer, colon cancer, glioblastoma, lung cancer, liver cancer, skin cancer, pancreatic cancer, and bladder cancer. Moreover, the different expression levels of mammalian plakins in many cancer types have been indicated by the Oncomine database. Mammalian plakins participate in cancer development and progression by regulating cell proliferation, migration, and invasion. To study the mechanisms, the altered cytoskeleton dynamics, along with certain signaling pathways such as Wnt/β-catenin, ERK1/2, and the PI3K/Akt axis have been shown to be involved. However, the exact mechanisms are still largely unknown. This is due to the fact that the available literature on the direct roles and associated mechanisms of plakins in human cancers is still scanty. Moreover, there are inconsistent findings on the role of some plakins in different cancers. Thus, more work needs to be conducted to answer the following questions: (1) What is the exact function of specific plakins in specific cancers? (2) What is the mechanism of specific plakins in specific cancer development and progression? (3) As most plakins have many different isoforms, is there a cancer-specific plakin isoform? (4) As our studies show the crucial function of MACF1 in osteoblasts and bone, are plakins involved in bone cancer? Answering these questions will increase the appeal of mammalian plakins to cancer research. Furthermore, the potential of mammalian plakins to be targets for cancer therapy makes them more attractive in future cancer research.

**Figure 4:**
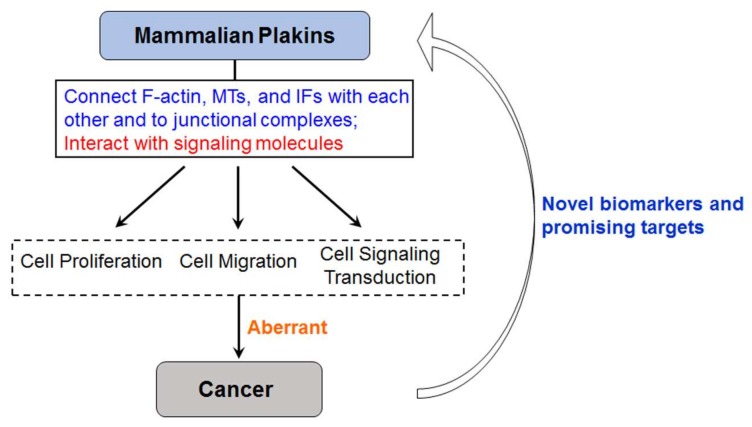
Mammalian plakins are novel biomarkers and promising targets for cancer diagnosis and therapy, given their versatile biological functions.

In summary, mammalian plakins, as giant cytolinkers that crosslink the three main cytoskeletal elements and junctional complexes, show versatility in regulating cell proliferation, migration, and signaling transduction. The alteration of mammalian plakins results in aberrant cell proliferation, migration, and signaling transduction, which causes cancer (Figure 4). Given their versatile biological functions, their critical roles in numerous cancers, and their potential to be targets for cancer therapy, mammalian plakins will therefore be novel biomarkers and promising targets for cancer diagnosis and therapy (Figure 4).

## Figures and Tables

**Table 1:** Mammalian plakin members, isoforms, intracellular localization, and tissue distribution.

Plakin Name (Gene)	Gene Locus (Human)	Isoforms	Molecular Weight (kD)	Intracellular Localization	Tissue Distribution
BPAG1 (*BPAG1*, *DST*)	Chromosome 6p12.1	BPAG1a1	~625	Cortical region, F-actin	Nervous system, brain, liver, spleen, ovary
		BPAG1a2		F-actin bundles surrounding the nucleus	Nervous system, brain, kidney, testis, liver, spleen, ovary
		BPAG1a3		Cortical region	Lung, kidney, testis, liver, spleen, ovary
		BPAG1b1	~834	Cortical region, F-actin	Heart, liver, spleen, ovary
		BPAG1b2		F-actin bundles surrounding the nucleus	Heart, testis, liver, spleen, ovary
		BPAG1b3		Cortical region	Lung, heart, testis, liver, spleen, ovary
		BPAG1e	~300	HDs, the nucleus	Epidermis, lung, testis, ovary, cornea, bladder
MACF1 (*MACF1*)	Chromosome 1p34.3	MACF1a1	~600	MTs, F-actin	Skin, kidney, stomach
		MACF1a2		MTs, F-actin	Brain, heart, lung placenta, liver, kidney, pancreas, spinal cord
		MACF1a3		MTs, F-actin	Skin, lung, kidney
		MACF1-4	670	Unclear	Heart, lung, pituitary gland, placenta
		MACF1b	~800	Golgi complex, MTs	Lung, heart, brain, thymus, liver, spleen, kidney, stomach, small intestine, skeletal muscle, skin, testis
		MACF1c	~600	Unclear	Nervous system
Plectin (*PLEC*)	Chromosome 8q24.3	Plectin 1	~500	Nucleus/ER membrane	Connective tissue, vascular, eye lens, skeletal muscle, white blood cells
		Plectin 1a		HDs	Broad
		Plectin 1b		Mitochondria	Connective tissue, skeletal muscle
		Plectin 1c		MTs	Broad
		Plectin 1d		Z-disks	Skeletal muscle
		Plectin 1f		FAs	Skeletal muscle
Desmoplakin (*DSP*)	Chromosome 6p24.3	Desmoplakin I	~322 kD	Desmosomes	Epithelia, heart
		Desmoplakin II	~259 kD	Desmosomes	Epithelia, heart
Envoplakin (*EVPL*)	Chromosome 17q25.1		210 kD	Cornified envelope	Stratified squamous epithelia
Periplakin (*PPL*)	Chromosome 16p13.3		195 kD	Cornified envelope, desmosomes	Stratified squamous epithelia
Epiplakin (*EPPK1*)	Chromosome 8q24.3		552 kD	Cornified envelope	Liver, small intestine, colon, salivary glands, stomach, appendix, pancreas, retina

BPAG1: bullous pemphigoid antigen 1; *DSP*: desmoplakin; *DST*: dystonin; *EPPK1*: epiplakin; ER: endoplasmic reticulum; *EVPL*: envoplakin; F-actin: actin filaments; FAs; focal adhesions; HDs: hemidesmosomes; MACF1: microtubule actin crosslinking factor 1; MTs: microtubules; *PLEC*: plectin; *PPL*: periplakin.

**Table 2:** Mammalian plakins in various cancers and the associated mechanisms.

Plakin Name	Cancer Type	Plakin’s Alteration and Functions	Mechanisms	References
BPAG1	Breast cancer	Downregulation, related to cancer invasion.	Unclear	[110]
	Nasopharyngeal carcinoma	Downregulation, related to tumor invasion and metastasis.	Unclear	[111]
	Prostate cancer	Downregulation, related to cancer metastasis.	Unclear	[112]
	OSCC	Positively regulates cell motility, invasion and tumorigenicity.	Upregulate NDRG1	[113,114]
	HNSCC	Alternative splicing revealed by expression microarray analysis.	Alternative splicing raises cancer-specific BPAG1 isoforms	[115]
MACF1	Lung cancer	High expression, related to cancer cell migration and metastasis. MACF1 knockdown impairs reproductivity of solid tumors.	Unclear	[116,117]
	Breast cancer	DNA methylation and altered expression, related to cell motility.	Unclear	[118,119]
	Renal cell carcinoma	Mutation.	Associated with Wnt/β-catenin signaling	[120]
	Endometrial cancer	Mutation.	Associated with Wnt/β-catenin signaling	[121]
	Colon cancer	Mutation.	Associated with Wnt/β-catenin signaling	[122]
	Glioblastoma	High expression, promotes cell proliferation and migration.	Activate Wnt signaling	[123]
	Hepatocellular carcinoma	A target of microRNA-34a.	Unclear	[124]
Plectin	Pancreatic IPMN	Positive expression.	Unclear	[127]
	Pancreatic cancer	Positive expression.	Unclear	[128]
	HNSCC	High expression, promotes cancer cell migration, invasion and metastasis.	Activates ERK1/2	[129]
	Colon cancer	High expression, promotes cancer cell migration, invasion and metastasis.	Be targeted to podosome-like adhesions in an isoform-specific manner	[130]
	Bladder cancer	High expression, promotes cancer cell migration and metastasis.	Anchor invadopodia to IF and stabilizing invadopodia	[131]
	OSCC	High expression, promotes cancer cell migration, invasion and tumorigenicity.	Upregulate NDRG1	[114]
	Liver cancer	Low expression, inhibits cancer cell migration.	Unclear	[132]
Desmoplakin	OSCC	Downregulation.	Unclear	[133]
	Oropharyngeal cancer	Downregulation.	Unclear	[134]
	Lung cancer	Downregulation, suppresses cell proliferation, migration and invasion, increases cancer sensitivity to anticancer drug-induced apoptosis.	Inhibits Wnt/β-catenin signaling	[135]
Envoplakin	Skin cancer	Promotes skin cancer development.	Unclear	[136]
Periplakin	Pharyngeal squamous cancer	Promotion effect on cell movement and attachment.	Activates the PI3K/Akt axis	[137]
	Urinary bladder cancer	Downregulation.	Unclear	[138]
	Esophageal squamous cancer	Downregulation, inhibits cell migration.	Mislocation of periplakin from cell–cell boundaries to cytoplasm; DNA hypermethylation	[139,140]
	Colon cancer	Downregulation, inhibits cell proliferation, migration, and invasion, induces G1/G0 cell cycle arrest.	Inhibits cell proliferation by suppressing ERK1/2 activation and PCNA expression	[141]
Epiplakin	Pancreatic cancer	Downregulation.	Unclear	[43]

BPAG1: bullous pemphigoid antigen 1; OSCC: oral squamous cell carcinoma; NDRG1: N-Myc downstream regulated gene 1; HNSCC: head and neck squamous cell carcinoma; MACF1: microtubule actin crosslinking factor 1; Wnt: wingless and int-1; IPMN: intraductal papillary mucinous neoplasms; ERK1/2: extracellular signal-regulated kinases 1/2; IF: intermediate filament; PI3K: phosphatidylinositol 3′ kinase; PCNA: proliferating cell nuclear antigen.

## Abbreviations

ABD	actin-binding domain
ACF7	actin crosslinking factor 7
AMPK	adenosine 5′-monophosphate-activated protein kinase
APC	adenomatous polyposis coli
BPAG1	bullous pemphigoid antigen 1
BPAG2	bullous pemphigoid antigen 2
BP230	bullous pemphigoid antigen 230
CAMSAP3	calmodulin regulated spectrin-associated protein 3
CCR	coiled-coil rod
CH	calponin homology
DSP	desmoplakin
ECM	extracellular matrix
ELMO	engulfment and motility
EPI−/−	triply deficient in envoplakin, periplakin, and involucrin
EPPK1	epiplakin
ER	endoplasmic reticulum
ERK1/2	extracellular signal-regulated kinases 1/2
EVPL	envoplakin
F-actin	actin filaments
FAK	focal adhesion kinase
FAs	focal adhesions
GAR	GAS2-related protein
Gas2	growth arrest specific 2
GSK3	glycogen synthase kinase 3
GSK-3β	glycogen synthase kinase 3β
GSR	glycine-serine-arginine
HD	hemidesmosome
HNSCC	head and neck squamous cell carcinoma
IFs	intermediate filaments
IPMN	intraductal papillary mucinous neoplasms
LRP5/6	low-density lipoprotein receptor 5/6
MACF1	microtubule actin crosslinking factor 1
MCH-1	melanin-concentrating hormone-1
MNK2	mitogen-activated protein kinase-interacting kinase 2
MTOC	microtubule organizing center
mTOR	mammalian target of rapamycin
MTs	microtubules
NDRG1	N-Myc downstream regulated gene 1
OSCC	oral squamous cell carcinoma
PCNA	proliferating cell nuclear antigen
PI3K	phosphatidylinositol 3′ kinase
PKA	protein kinase A
PKB	protein kinase B
PKC	protein kinase C
PLEC	plecitn
PPL	periplakin
PRD	plakin repeat domain
RCSB	research collaboratory for structural bioinformatics
SH3	src-homology 3
Sp1	specificity protein 1
TCF	T-cell factor
UTRs	untranslated regions
Wnt	wingless and int-1
